# Health insurance coverage, medical expenditure and coping strategy: evidence from Taiwan

**DOI:** 10.1186/1472-6963-12-442

**Published:** 2012-12-03

**Authors:** Kuangnan Fang, Ben-Chang Shia, Shuangge Ma

**Affiliations:** 1 Department of Statistics, Xiamen University, Xiamen, China; 2 Department of Statistics and Information Science, FuJen Catholic University, Taiwan, R.O.China; 3 School of Public Health, Yale University, 60 College ST, New Haven, CT, 06520, USA

**Keywords:** Taiwan, Health insurance coverage, Medical expenditure, Coping strategy

## Abstract

**Background:**

The health insurance system in Taiwan is comprised of public health insurance and private health insurance. The public health insurance, called “universal national health insurance” (NHI), was first established in 1995 and amended in 2011. The goal of this study is to provide an updated description of several important aspects of health insurance in Taiwan. Of special interest are household insurance coverage, medical expenditures (both gross and out-of-pocket), and coping strategies.

**Methods:**

Data was collected via a phone call survey conducted in August and September of 2011. A household was the unit for survey and data analysis. A total of 2,424 households covering all major counties and cities in Taiwan were surveyed.

**Results:**

The survey revealed that households with smaller sizes and higher incomes were more likely to have higher coverage of public and private health insurance. In addition, households with the presence of chronic diseases were more likely to have both types of insurance. Analysis of both gross and out-of-pocket medical expenditure was conducted. It was suggested that health insurance could not fully remove the financial burden caused by illness. The presence of chronic disease and inpatient treatment were significantly associated with higher gross and out-of-pocket medical expenditure. In addition, the presence of inpatient treatment was significantly associated with extremely high medical expenditure. Regional differences were also observed, with households in the northern, central, and southern regions having less gross medical expenditures than those on the offshore islands. Households with the presence of inpatient treatment were more likely to cope with medical expenditure using means other than salaries.

**Conclusion:**

Despite the considerable achievements of the health insurance system in Taiwan, there is still room for improvement. This study investigated coverage, cost, and coping strategies and may be informative to stakeholders of both basic and commercial health insurance.

## Background

The health insurance system in Taiwan is composed of public and private health insurance. The public insurance system, called “universal national health insurance” (NHI), was inaugurated in 1995 and administrated by the Department of Health. The NHI aimed to ensure that all Taiwanese people had access to health care, regardless of their financial status. However, in order to avoid moral hazard, patients were required to make copayments when they received outpatient or inpatient care, dental care, emergency care, or Chinese medicine services. The copayments gave patients incentives to limit their medical visits and thus reduce medical costs. Occasionally, the medical costs could be considerably high. Copayments varied according to the type of provider. For example, medical centers charged more than clinics for outpatient care [1]. To further reduce potential financial losses caused by illness, people also purchased private health insurance. By 2010, Taiwan’s private health insurance market was composed of twenty-three domestic and seven foreign companies [2]. Interestingly, no significant conflict was observed between the public and private insurance systems. Taiwan experienced growth in the demand for private health insurance with the NHI. Possible explanations were offered by Liu and Chen [3].

There has been extensive research conducted on Taiwan’s health care and health insurance system. Cheng provided descriptions of several major aspects of the NHI, including government administration, access, freedom in choice, utilization of services, and benefits offered [4]. Studies such as [5] discussed the financial burden for both the government and the insured. There were also studies focusing on women, children, and the elderly, who were not covered by public insurance before the implementation of the NHI [6,7]. Wen and others analyzed the role of the NHI in improving life expectancy and reducing health disparities [8,9]. The differences between different economic groups and between rural and urban areas were also of interest [10,11]. Most published studies, including those mentioned above, focused on the NHI, which was only one side of the health insurance system, with the other side being private insurance. Studies on the NHI had investigated multiple perspectives, including, for example, access, expenditure, and copayment, and demonstrated that the NHI had significant effects on the insured.

In this study, we focused on the coverage, expenditure and coping aspects of health insurance in Taiwan, with the target being providing a relatively comprehensive description of the health insurance system there. Regarding coverage and access, Lu and Hsiao showed that, by the end of 2001, 97% of the total eligible population had enrolled in the NHI [5]. Lee and others reported a coverage rate over 98% [11]. It is worth noting that this data had been generated by the Bureau of National Health Insurance, as opposed to third-party independent research. Regarding cost, Cheng and Chiang concluded that the coping expenditure mechanism designed in the insurance scheme seemed to have an insignificant effect on curbing medical care utilization [12]. Lu and Hsiao illustrated that the population covered by the NHI was well protected against large medical expenses, and the gross medical cost was under control [5]. Despite the significant implications of the NHI, it was only a part of the health insurance system. For the insured, it can be difficult to separate public insurance from private insurance. To provide a more comprehensive description, both types of insurance need to be considered. In addition, both the private and public health insurance sectors are still undergoing fast evolvement. Thus, there is a need for a timely update. The objective of this study was to provide a comprehensive and updated description of several important aspects of both the public and private health insurance sectors in Taiwan.

## Methods

**Figure 1:**
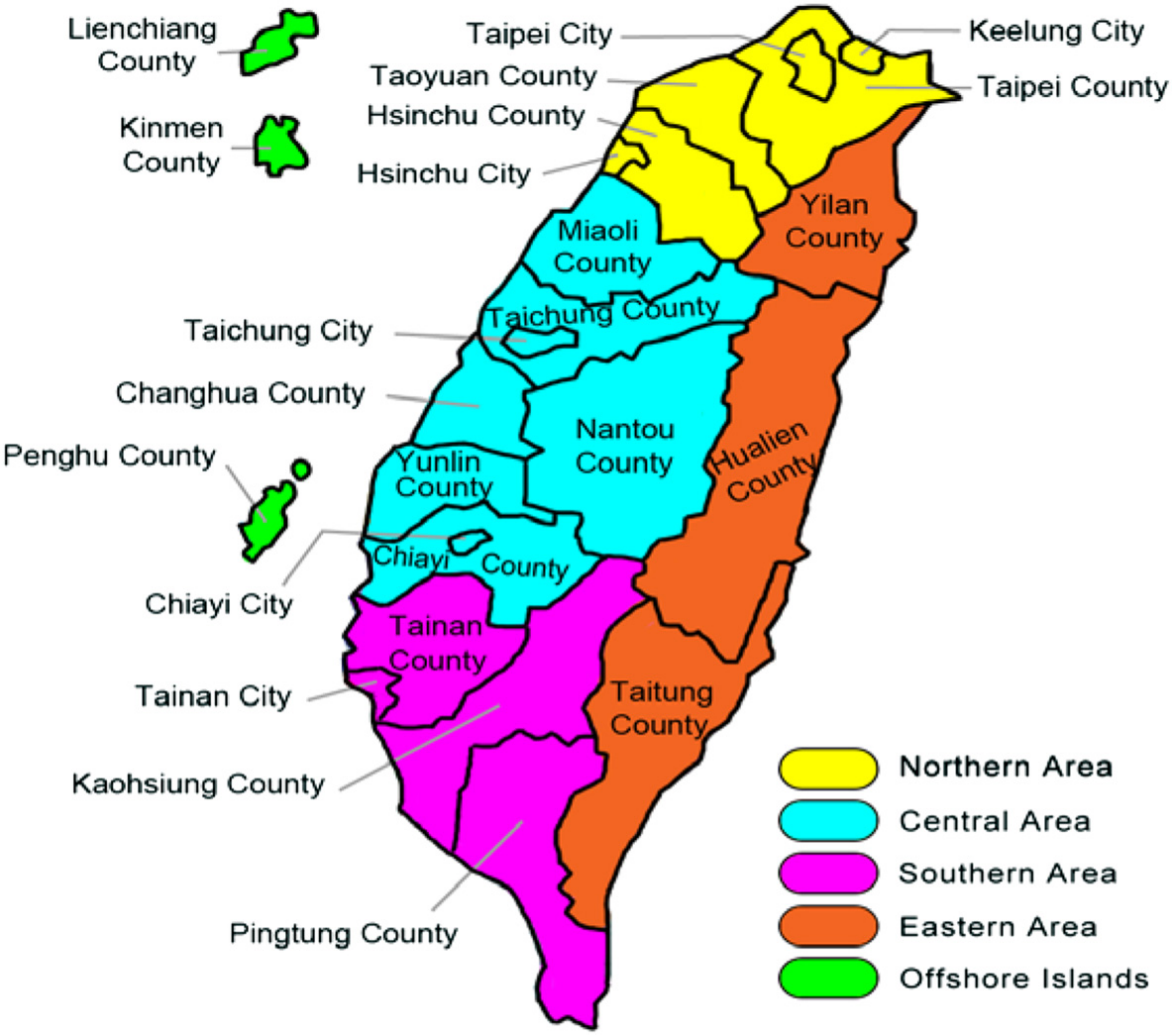
Map of Taiwan.

Taiwan is located in East Asia, has a land area of 36,000 square kilometers, and has a population of 23 million. The per capita GDP, based on purchasing power parity (PPP), was predicted to be $35,604 in 2011. In Taiwan, there are eighteen major counties and cities and Taipei, Kaohsiung, Xinbei, Taichung, and Tainan, which are under the direct administration of the central government. Based on the geographic location, they have been separated into the northern, central, southern, eastern, and offshore regions (Figure 1).

The study protocol was reviewed and approved by the Research Ethics Review Committee at FuJen Catholic University in Taiwan. The administration of the study was also monitored by the same committee. Data was collected in a cross-sectional survey conducted in August and September of 2011. The unit of sampling was household. The phone call survey was conducted by staff at the Department of Statistics and Information Science at FuJen Catholic University. An RDD (random digit dialing) method was used to select samples. Mitofsky-Waksberg type samples [13] of active blocks of one hundred consecutive telephone numbers from all possible blocks within each county (city) were drawn. The probability of a block’s initial selection was a positive linear function of the proportion of the block’s one hundred numbers that served residences. In the sample selection, we focused on landlines only, as it was difficult to associate a cell phone number with a physical location for a household. Cell phones are becoming increasingly popular in Taiwan, and it is expected that there are a certain number of “cell phone only” households. In the literature, there is a lack of study comparing households with and without landlines. Focusing on landlines may lead to a biased sample. However, such a bias is not expected to be substantial.

At the beginning of each survey, the staff introduced the purpose of the survey. Follow-up questions were not asked if the interviewee was under eighteen or could not provide reliable information on the household. Verbal consent was obtained from each interviewee and recorded using a voice-recording software package. Information was gathered using a structured questionnaire, which included questions on demographics, health condition, insurance coverage, medical expenditure, and coping strategies. For each question, the survey staff offered a set of predefined options. Some questions, such as household size and insurance coverage, were “snapshots” at the time of the survey. Other questions, such as household income, consumption of health care, health condition, and coping strategies, were designed to reflect accumulation over a period of twelve months. The survey response rate was about 48%.

S-Plus version 8.2 (TIBCO Software Inc.) was used for analysis. ANOVA and Chi-square tests were used to examine differences among data collected in different counties and cities. Although some differences were observed, it was determined to be sensible to combine the data and analyze. Summary statistics were computed for the whole cohort and subgroups, and between-group comparisons were conducted using t-tests, Chi-square tests, and Fisher’s exact tests. As the dependent variables of main interest were categorical due to the nature of the survey, a logistic regression analysis was conducted. For coverage, each household’s coverage rate was computed as the number of people covered divided by household size. Because of the significant differences between the NHI and private insurance, their coverage rates were also analyzed separately. For all analyses, coverage rates were dichotomized at 50% to create dummy variables. For medical expenditure, two different measures were analyzed. One was gross medical expenditure, and the other was out-of-pocket expenditure. For coping, strategies for dealing with high and extremely high cost were analyzed separately.

## Results

### Descriptive statistics

Descriptive statistics of the 2,424 households with 9,593 members are shown in Table 1. A total of 91.63% of the members were covered by public health insurance. The rate was lower than that reported by Lu and Hsiao [5]. The samples were split into two groups based on whether insurance coverage was greater than 50%. Out of the 2,424 households, 96.9% (2,349) had more than half of the household members covered. 96.5% (2,347) and 10.6% (257) of the households had more than half of the members covered by public and private health insurance, respectively. There were 1,001 households from the northern area, 702 from the central area, 444 from the southern area, 103 from the eastern area, and 174 from the offshore islands. The average household size of the whole cohort was 3.957. The averages of household size were 4.014 and 2.173 for the high and low overall insurance coverage groups, respectively (p-value <0.001). Similarly, households with higher public insurance coverage and private insurance coverage had smaller sizes (both p-values<0.001). There was a significant difference in income between high and low private insurance coverage groups (p-value <0.001). Particularly, households with higher income tended to have more private insurance coverage. Since household expense was correlated with income, a similar association was observed between coverage rate and expense. In this study, health condition was measured by the number of hospitalized inpatient treatment and the presence of diagnosed chronic disease(s). About 30% of the households with family members had at least one inpatient treatment. Households with higher coverage rates were more likely to have five or more inpatient treatments. For example, 14.67% of the households in the high overall coverage group had five or more inpatient treatments, compared to only 0.81% in the low coverage group (p-value<0.001). There was no significant association between the presence of chronic disease(s) and coverage.

**Table 1:** Summary statistics of the whole sample and stratified by insurance status

**Variables**	**Total**	**Combined insurance coverage**		**Public insurance coverage**		**Private insurance coverage**	
		**>50%**	**=<50%**	**>50%**	**=<50%**	**>50%**	**=<50%**
**Area**							
Whole cohort	2424	2349	75	2347	77	257	2167
Northern area	1001	969	32	968	33	101	900
Central area	702	678	24	677	25	74	628
Southern area	444	428	16	428	16	48	396
Eastern area	103	101	2	101	2	10	93
Offshore islands	174	173	1	173	1	24	150
p-value		(<0.001)		(<0.001)		(0.688)	
**Household size**	3.957	4.014	2.173	4.140	2.220	4.595	3.882
Mean (sd)	(1.711)	(1.706)	(0.381)	(1.769)	(0.476)	(2.042)	(1.651)
P- value		(<0.001)		(<0.001)		(<0.001)	
**Household income** (**Percentage**)							
Less than 135,000	23.47	23.41	25.33	23.43	24.68	18.68	24.04
135,000-225,000	18.44	18.18	26.67	18.19	25.97	6.61	19.84
225,000-450,000	25.04	25.03	25.33	25.05	24.68	22.18	25.38
450,000-675,000	18.56	18.82	10.67	18.75	12.99	25.68	17.72
More than 6750,000	14.48	14.56	12.00	14.57	11.69	26.85	13.01
P- value		(0.207)		(0.372)		(<0.001)	
**Household expense** (**Percentage**)							
Less than 45,000	11.06	11.15	8.00	11.16	7.79	5.45	11.72
45,000-135,000	25.41	25.37	26.67	25.39	25.97	15.18	26.63
135,000-225,000	36.63	36.82	30.67	36.77	32.47	22.18	38.35
225,000-450,000	18.56	18.48	21.33	18.49	20.78	33.46	16.80
More than 450,000	8.33	8.17	13.33	8.18	12.99	23.74	6.51
P- value		(0.393)		(0.488)		(<0.001)	
**Number of inpatient treatment** (**Percentage**)							
None	69.22	69.60	57.33	69.58	58.44	78.21	68.16
One	20.50	20.43	22.67	20.45	22.08	21.79	20.35
Two	7.47	7.54	5.33	7.54	5.19	0	8.35
Three	1.49	1.53	0	1.53	0	0	1.66
Four	0.08	0.09	0	0.09	0	0	0.09
Five or more	1.24	0.81	14.67	0.81	14.29	0	1.38
P-value		(<0.001)		(<0.001)		(<0.001)	
**Presence of chronic disease** (**Percentage**)							
Yes	36.63	36.61	37.33	36.64	36.36	33.07	37.06
No	63.37	63.39	62.67	63.36	63.64	66.93	62.94
P-value		(0.995)		(0.944)		(0.236)	

### Insurance coverage

The results of the coverage regression analysis are shown in Table 2. Household size had a significant effect on both public and private insurance coverage, with odds ratios of 0.84 and 0.26, respectively, indicating that bigger households were more likely to have lower insurance coverage. This result differed from that of an earlier study [3]. High income levels (between NT$450K and NT$675K and over NT$675K) had a positive effect on public and private insurance coverage (odds ratios of 2.921 and 2.542, 1.234 and 1.131, respectively), suggesting that households with higher incomes were more likely to have private and public insurance. Such a finding was consistent with Liu and Chen [3]. Studies on health insurance in Western countries also suggested an association between health insurance coverage and income [14,15]. There was a significant association between expense and insurance coverage, with high expense (>NT$450K) having a significantly negative effect on public insurance coverage (odds ratio of 0.182). High expense levels had a positive effect on private insurance coverage (odds ratios of 4.158 and 7.099). Households with higher expenses had higher incomes and were more likely to be able to afford private insurance. The presence of chronic disease(s) was associated with a higher coverage rate. Chronic diseases are long-lasting, allowing households to obtain insurance to cope with future medical expenses. The presence of inpatient treatment had negative effects on both public and private insurance coverage (odds ratios of 0.499, 0.529, and 0.596, respectively). For both public and private insurance coverage, there was no significant difference among regions. This finding differed from that in [3], which showed a dependence of coverage on residential location.

**Table 2:** Logistic regression results of insurance coverage

**Variables**	**Public insurance**	**Private insurance**
**Household size**	0.84^***^	0.26^***^
**Household income** (**baseline**: <**135K**)		
B: between 135K and 225K	0.825	0.424***
C: between 225K and 450K	1.493	0.940
D: between 450K and 675K	2.542**	1.234**
E: over 675K	2.906**	1.131**
**Household expense** (**baseline**: <**45K**)		
B: between 45K and 135K	0.722	1.647
C: between 135K and 225K	0.562	1.351
D: between 225K and 450K	0.366	4.158***
E: over 450K	0.182***	7.099***
**Presence of chronic disease**	1.169**	1.150 ***
**Presence of inpatient treatment**	0.529**	0.596***
**Regions** (**Offshore islands as baseline**)		
Northern area	0.144	0.959
Central area	0.144	0.894
Southern area	0.155	0.873
Eastern area	0.259	0.943

*Note*: Numbers presented are odds ratios. *** statistically significant at 1%; **statistically significant at 5%. *statistically significant at 10%.

Increasing coverage is an important goal for both the public and private insurance sectors. The NHI was designed to provide universal coverage. The coverage rate observed in this survey was lower than that reported by the Taiwanese central government. The above analysis identified features of the subgroups with lower coverage, which should be the target to improve coverage. Tuning public policies to achieve increased coverage has been investigated in multiple published studies and will not be discussed here.

### Gross and out-of-pocket medical expenditure

Both gross and out-of-pocket medical expenditure were analyzed. Gross medical expenditure could be of significant interest to the government and insurance companies, while out-of-pocket medical expenditure was more important to measure real household expenditure. Published studies have shown that out-of-pocket medical expenditure was an important factor for poverty in many Asian countries [16]. The insured poor faced financial barriers to health care services, and their illness conditions had a negative effect on their financial situations [2]. For each type of medical expenditure, a categorical response variable was created, which was easier to manage in the survey and less likely to be subject to recall error than a continuous variable. Households were split in two groups, medical expenditures >NT$45K versus <=NT$45K. As the response variables were binary, logistic models were used. Analysis results are shown in Table 3.

**Table 3:** **Logistic regression results of gross and out**-**of**-**pocket medical expenditure**

**Variables**	**Gross medical expenditure>45K**	**Out**-**of**-**pocket medical expenditure>45k**
**Household size**	1.258 ***	1.190***
**Household income** (**baseline**: <**135K**)		
B: between 135K and 225K	0.649	0.617*
C: between 225K and 450K	0.354	0.317***
D: between 450K and 675K	0.406**	0.403 ***
E: over 675K	1.501 ***	1.249 ***
**Presence of chronic disease**	1.253	1.296
**Presence of inpatient treatment**	3.611***	3.651***
**Public insurance coverage**	0.760	2.170
**Private insurance coverage**	1.602	7.381 **
**Regions**(**Offshore islands as baseline**)		
Northern area	0.665	0.706
Central area	0.511 *	0.551
Southern area	0.751	0.806
Eastern area	0.997	1.069

*Note*: Numbers presented are odds ratios. ***statistically significant at 1%; **statistically significant at 5%; *statistically significant at 10%

The low and moderate cost group (<=NT$45K) was compared against the extremely high cost group (>NT$45K). Household size had a significant positive effect on out-of-pocket expenditure, with an odds ratio of 1.190. All income levels had significant associations with out-of-pocket medical expenditure. The low and medium income levels had negative effects, whereas the high income group (>NT$675K) was more likely to have higher out-of-pocket medical expenditure, which was consistent with published studies. For example, Chu and others [2] found that average out-of-pocket expenditure increased in conjunction with increased income. Inpatient treatment had a significant effect on out-of-pocket medical expenditure, with an odds ratio of 3.651 (p-value<0.001). However, the association between the presence of chronic disease(s) and out-of-pocket medical expenditure was not significant. Private insurance coverage had a strong effect (an odds ratio of 7.381), while the effect of public insurance coverage was not significant. Liu and Chen [3] suggested that the higher the copayment, the more likely households were to buy private health insurance to protect themselves against potential catastrophic financial burdens. There was not a significant difference among regions.

### Coping strategies

Our analysis suggested a considerable amount of out-of-pocket medical expenditure. It was thus of interest to investigate coping strategies – ways that households paid for medical expenses. In the survey, there were five options for coping strategies, including (A) salary from last month, (B) saving, (C) help from family and friends, (D) loan, and (E) reduction in daily living cost. The distribution of coping strategies A-E was 64.65% (1,567), 28.84% (699), 0.7% (17), 1.03% (25) and 4.78% (116), respectively. The majority of the households were able to self-finance out-of-pocket costs (answers A and B). Here, two sets of analysis were conducted. In the first set, strategy A was compared against B-E. Covering medical costs using last month’s salary was the “best” coping strategy, imposing the least long-term impact. In the second set of analysis, strategies A-B were compared against C-E, as options A and B corresponded to self-finance. As the coping strategy outcomes were binary, logistic regressions were conducted. The analysis results are shown in Table 4.

**Table 4:** Logistic regression results of coping strategies

**Variables**	**Salary**	**Salary** + **Saving**
**Household size**	1.091 ***	0.255***
**House income** (**baseline**: <**135K**)		
B: between 135K and 225K	0.569 ***	0.490***
C: between 225K and 450K	0.602 ***	0.197***
D: between 450K and 675K	1.033	0.518***
E: over 675K	0.619 ***	0.127***
**Presence of chronic disease**	0.882	1.622 ***
**Presence of inpatient treatment**	1.317***	1.866 ***
**Public insurance coverage**	0.655	0.066 ***
**Private insurance coverage**	0.861	0.025 ***
**Regions** (**Offshore islands as baseline**)		
Northern area	0.983	0.985
Central area	1.017	1.176
Southern area	1.034	1.144
Eastern area	0.928	0.769

*Note*: Numbers presented are “odds ratio”. ***statistically significant at 1%, **statistically significant at 5%. *statistically significant at 10%. In the first regression, the outcome variable “using salary (A)” was coded as 0, and “using other means (B-E)” was coded as 1. In the second regression, the outcome variable “salary or saving (A-B)” was coded as 0, and “other means (C-E)” was coded as 1.

In the first set of analysis, household size was found to have a significant effect on coping strategy (an odds ratio of 1.091). Income levels, except for the level between NT$450K and NT$675K, had significant effects, with those with higher income levels less likely to use means other than salary (odds ratios of 0.569, 0.602, and 0.619, respectively). Inpatient treatment had a significant effect on coping strategy (an odds ratio of 1.317), which implied that households with inpatient treatments were more likely to use savings, loans and other means. However, the association between the presence of chronic disease(s) and coping strategies was not significant. Inpatient treatments happened with low frequencies and hit households without warning. As it was difficult to plan for such incidents ahead of time, households were more likely to pursue coping strategies other than salary. Chronic diseases were recurrent, with low to moderate costs per episode. Well-planned households could have well-adjusted coping plans that covered costs using monthly income without having to resort to outside financial sources. The effects of public and private insurance coverage were not significant, and there was no significant difference among regions.

In the second set of analysis, household size had a significant effect on coping strategy. All income levels had significant associations with coping strategies, and the odds ratios were smaller than one (0.49, 0.197, 0.518, and 0.127, respectively), suggesting that the higher the income, the less likely were households to pay using means other than salary and savings. The effects of the presence of chronic disease(s) and inpatient treatments were significant (odds ratios of 1.622 and 1.866, respectively). Unlike in the first set of analysis, public and private insurance coverage had significant associations with coping strategies (odds ratios of 0.066 and 0.025, respectively), suggesting that the higher the public and private insurance coverage, the less likely were households to pursue coping strategies other than salary and savings.

Most of the surveyed households were able to self-finance out-of-pocket medical expenses using salary and savings, without having to rely on outside financial sources or reduce daily living costs. However, there were still 6% of the households that warranted further attention. Future policy development may focus on this subcohort, who may suffer a longer financial impact caused by illness and out-of-pocket expenditure.

## Discussion and conclusion

In this article, we reported on a survey recently conducted on health insurance in Taiwan. This study complements published ones by providing comprehensive, updated descriptions of several important aspects of Taiwan’s health insurance arena. The health insurance system in Taiwan has been appraised as being one of the successful models in existence. Understanding its achievements as well as its problems may provide valuable information to various Taiwanese insurance agencies and, more importantly, to countries that are or will be pursuing similar systems.

For both the public and private insurance sectors, increasing coverage is an important goal. Particularly for the NHI, the ultimate goal is to cover the whole population. Households with smaller sizes and higher income levels (an annual income greater than NT$450K) were more likely to buy private and public insurance coverage. Households with the presence of chronic diseases were more likely to have public and private health insurance coverage. It is of interest to note the negative association between coverage rate and inpatient treatment. Cost associated with inpatient treatment is an important component of catastrophic health expenditure [17,18], which may directly lead to poverty. From a policy point of view, it is of significant interest to design the insurance system in a way that can protect such households. Two kinds of medical expenditure, gross and out-of-pocket, were investigated. Gross medical expenditure can be of significant interest to government agencies, while out-of-pocket medical expenditure can measure the real expenditure of a household on health care. Our study found that health insurance could not completely remove the financial burden caused by illness. This study also investigated coping strategies. Households with family members having inpatient treatments in the past twelve months were more likely to use savings, loans and other means different from salary. However, the association between the presence of chronic disease(s) and coping strategies was not statistically significant. Making financial decisions is a very complicated process. Factors investigated in this study are far from complete. Observations made in this study can be informative, however, should be interpreted with extreme caution.

Investigating both public and private insurance provides a more comprehensive description of households’ insurance status. However, a tradeoff is that the policy implications of the study results may be ambiguous as the net effect of public insurance could not be investigated. It is thus not clear how the government should tune the public insurance policy. Because of the phone call survey nature of this study, the collected information might not be detailed enough. Particularly, the data were either snapshots at the time of the survey or an aggregation over twelve months. Such data had limitations. For example, insurance status and household size might change over time. The aggregated data could not describe the variations across different illness episodes and their differences in financial consequences. In addition, it had been suggested that measuring out-of-pocket cost as a single item might result in a slightly biased estimation (usually under-estimation). On the other hand, the present design also has advantages. Specifically, households often had multiple illness episodes, and people tended to remember the total cost and how they paid for all of them in general, rather than for a single episode. It was possible or even likely that multiple coping strategies were used, while the survey focused on the single most important coping strategy. The aforementioned limitations are also shared by studies of a similar nature.

## Competing interests

The authors declare that they have no competing interests.

## Authors’ contributions

SM designed the study. KF and BS conducted the study. KF conducted statistical analysis. SM drafted the manuscript with input from KF and BS. All authors read and approved the final manuscript.

## Pre-publication history

The pre-publication history for this paper can be accessed here:

http://www.biomedcentral.com/1472-6963/12/442/prepub
